# Associations of PGK1 promoter hypomethylation and PGK1-mediated PDHK1 phosphorylation with cancer stage and prognosis: a TCGA pan-cancer analysis

**DOI:** 10.1186/s40880-019-0401-9

**Published:** 2019-10-02

**Authors:** Fei Shao, Xueying Yang, Wei Wang, Juhong Wang, Wei Guo, Xiaoli Feng, Susheng Shi, Qi Xue, Shugeng Gao, Yibo Gao, Zhimin Lu, Jie He

**Affiliations:** 1 Department of Thoracic Surgery, National Cancer Center/National Clinical Research Center for Cancer/Cancer Hospital, Chinese Academy of Medical Sciences and Peking Union Medical College, No.17 Panjiayuannanli, Chaoyang District, Beijing, 100021 P. R. China; 2 Qingdao Cancer Institute, Cancer Institute of The Affiliated Hospital of Qingdao University, Qingdao, 266003 Shandong P. R. China; 3 Zhejiang Provincial Key Laboratory of Pancreatic Disease, The First Affiliated Hospital, and Institute of Translational Medicine, Zhejiang University School of Medicine, Hangzhou, 310029 Zhejiang P. R. China; 4 Department of Pathology, National Cancer Center/National Clinical Research Center for Cancer/Cancer Hospital, Chinese Academy of Medical Sciences and Peking Union Medical College, Beijing, 100021 P. R. China; 5 State Key Laboratory of Molecular Oncology, National Cancer Center/National Clinical Research Center for Cancer/Cancer Hospital, Chinese Academy of Medical Sciences and Peking Union Medical College, Beijing, 100021 P. R. China

**Keywords:** PGK1, Cancer metabolism, Epigenetics, Phosphorylation, Methylation, The Cancer Genome Atlas, Pan-cancer analysis, Prognosis, Overall survival

## Abstract

**Background:**

Cancer cells reprogram metabolism for proliferation. Phosphoglycerate kinase 1 (PGK1), as a glycolytic enzyme and newly identified protein kinase, coordinates glycolysis and mitochondrial metabolism. However, the clinical significance of PGK1 expression and function in cancer progression is unclear. Here, we investigated the relationship between the progression and prognosis of multiple cancer types and PGK1 expression and its function in the mitochondrial metabolism regulation.

**Methods:**

We performed pan-cancer analyses of *PGK1* mRNA level and DNA methylation in 11,908 tumor tissues and 1582 paired normal tissues across 34 cancer types in The Cancer Genome Atlas datasets. Using specific antibodies against PGK1 S203 and PDHK1 T338 phosphorylation, we performed immunohistochemistry with tissue microarray assay in additional 818 cancer cases with 619 paired normal tissues from five cancer types.

**Results:**

The *PGK1* mRNA level was significantly elevated with hypomethylation in promotor regions and associated with advanced TNM stage in 15 and four cancer types, respectively. In breast carcinoma, elevated *PGK1* mRNA level and promoter hypomethylation were associated with poor prognosis. Positively correlated PGK1 S203 and PDHK1 T338 phosphorylation levels were significantly associated with short overall survival (OS) in cancers of the breast, liver, lung, stomach, and esophagus and with advanced TNM stage in breast and esophageal cancers. PGK1 pS203 and PDHK1 pT338 were also independent predictors of short OS in liver, lung, and stomach cancer.

**Conclusions:**

The elevated expression, promoter hypomethylation, and phosphorylation of PGK1 and PDHK1 were related with disease progression and short OS in diverse types of cancer. PGK1 and PDHK1 phosphorylation may be potential prognostic biomarkers.

## Background

Most cancer cells, even in the presence of ample oxygen, predominantly generate adenosine triphosphate (ATP) by a high rate of glycolysis followed by lactate fermentation in the cytosol rather than by oxidation of pyruvate in the mitochondria, as in most normal cells. This phenomenon, known as *aerobic glycolysis* or the *Warburg effect*, facilitates tumor cell growth [1–5]. In the glycolytic pathway, phosphoglycerate kinase 1 (PGK1), the first enzyme to generate ATP, catalyzes the transfer of the high-energy phosphate from 1,3-diphosphoglycerate to adenosine diphosphate (ADP), leading to the generation of 3-phosphoglycerate and ATP. In *Homo sapiens*, PGK has two isozymes, ubiquitously expressed PGK1 and testis-expressed PGK2, both with 87%–88% identical amino acid sequence identity [6].

The reprogramming of metabolism is an emerging hallmark of cancer biology [4, 7, 8]. Recent studies have shown that the protein level of PGK1 was elevated in breast cancer [9], astrocytoma [10], metastatic colon cancer [11], and pancreatic ductal adenocarcinoma [12]; its mRNA levels were increased in gastric cancer [13].

Our previous studies [14] revealed that in tumor cells, PGK1 possesses protein kinase activity in addition to performing its well-established glycolytic function. In response to receptor tyrosine kinase activation, the expression of K-Ras G12V and B-Raf V600E, hypoxia, pyruvate metabolism in mitochondria is suppressed [14, 15]. This is primarily regulated by the mitochondrial translocation of PGK1, which is phosphorylated at S203 by extracellular signal-regulated kinase 1/2 (ERK1/2) and *cis*–*trans* isomerized by peptidyl-prolyl *cis*–*trans* isomerase NIMA-interacting 1 (PIN1), leading to exposure of the pre-sequence of PGK1 for binding to the translocase of the outer membrane (TOM) complex of mitochondria. In the mitochondria, PGK1 functions as a protein kinase to phosphorylate pyruvate dehydrogenase kinase 1 (PDHK1, also known as PDK1) at T338, which activates PDHK1 to phosphorylate and inhibit the pyruvate dehydrogenase (PDH) complex [14, 15]. Suppression of PDH activity reduces mitochondrial pyruvate utilization and reactive oxygen species production and increases lactate production, thereby promoting tumorigenesis. In addition, PGK1 S203 and PDHK1 T338 phosphorylation levels were found to be positively correlated with each other, and both were correlated with PDH S293 inactivating phosphorylation levels and poor prognosis in patients with glioblastoma (GBM) [14]. However, whether the newly identified protein kinase function of PGK1 applies to other cancer types and the relationship between PGK1 kinase activity and tumor progression remain unknown.

Here, we performed a pan-cancer analysis of clinical relevance of *PGK1* using data from 11,908 cases (including 1582 with paired normal tissues) across 34 cancer types from The Cancer Genome Atlas (TCGA) datasets. We also analyzed the clinical relevance of PGK1 S203 and PDHK1 T338 phosphorylation levels by conducting immunohistochemical experiments in an additional 818 independent cancer cases (including 619 with paired normal tissues). We aimed to evaluate the pathological progression value and prognostic values of *PGK1* mRNA high expression, *PGK1* promoter methylation, and PGK1 mediated-PDHK1 activating phosphorylation in multiple human cancers.

## Materials and methods

### Data resource

We downloaded clinical records, RNAseqV2 level 3 gene level data, and DNA methylation level 3 data for 11,908 cases across 34 cancer types from TCGA (http://xena.ucsc.edu/welcome-to-ucsc-xena/). Profiling data of the TCGA-retrieved cases were generated using the Illumina HiSeq 2000 RNA Sequencing and Illumina Infinium Human Methylation 450 platforms, as described by the TCGA network [16, 17]. Gene transcription estimates for each gene were presented as in RNA-Seq using the Expectation Maximization (RSEM) software. DNA methylation values are presented as beta values for each CpG probe transformed into M values. The detailed information about data processing is provided in Additional file 1: Methods. A summary of the sample sizes for the *PGK1* RNA-Seq and DNA methylation analyses for each cancer type is shown in Additional file 1: Table S1. There are 16 methylation probes that cover the *PGK1* gene (Chromosome X; UCSC Gene Accession: NM_000291) (Additional file 1: Table S2).

The histopathologic diagnoses of the TCGA cases are available in the Genomic Data Commons (GDC, https://portal.gdc.cancer.gov/).

*PGK* gene level data in 16 tissue types were downloaded from the Illumina Body Map Project (https://www.ebi.ac.uk/gxa/home), and the results are presented as transcripts per million (TPM) values.

### Patients and tissue samples

We retrospectively collected surgically resected, formalin-fixed, paraffin-embedded tissue samples from the biobank of National Cancer Center/National Clinical Research Center for Cancer/Cancer Hospital in Chinese Academy of Medical Sciences and Peking Union Medical College (Beijing, China). Tissue samples of 818 treatment-naïve patients who underwent surgery for pathologically diagnosed cancer between 2006 and 2015 were selected as an additional independent cohort, including 145 cases of breast carcinoma (BRCA) (with 69 paired normal specimens), 185 cases of liver hepatocellular carcinoma (LIHC) (with 174 paired normal specimens), 179 cases of lung adenocarcinoma (LUAD) (with 175 paired normal specimens), 95 cases of stomach adenocarcinoma (STAD) (with 55 paired normal specimens), and 214 cases of esophageal carcinoma (ESCA) (with 146 paired normal specimens).

### Tissue microarray construction

Rabbit polyclonal antibodies recognizing phospho-PGK1 S203 and phospho-PDHK1 T338 were obtained from Signalway Antibody (College Park, MD, USA). The specificities of these antibodies were previously validated [14]. A rabbit monoclonal antibody recognizing IgG was purchased from Cell Signaling Technology (Danvers, MA, USA). Formalin-fixed, paraffin-embedded tissues were obtained by surgical resection, archived after clinical use for pathological diagnosis, and stained with Mayer’s haematoxylin and eosin (H&E; Biogenex Laboratories, San Ramon, CA, USA).

Tumor samples from the 818 cancer cases with 550 paired normal tissues were subjected to tissue microarray (TMA). Employing an automated tissue array instrument (Minicore^®^ 3, Alphelys, Plaisir, France), cancer tissue (diameter at 2 mm, selected by a pathologist) from each specimen was extracted and fixed into a paraffin block. After quality control, the TMA blocks were sectioned into 3-μm-thick slides for immunohistochemistry analysis.

### Immunohistochemistry

After deparaffinization, rehydration, and antigen-retrieval, TMA slides were incubated with primary rabbit anti-human phospho-PGK1 S203 (dilution 1:200; Signalway Antibody; SAB487P), primary rabbit anti-human phospho-PDHK1 T338 (dilution 1:500; Signalway Antibody; #11596), or nonspecific IgG (as a negative control) overnight at 4 °C. The slides were then incubated with anti-rabbit secondary antibody (ready-to-use solution; Cell Signaling Technology; #8114), followed by chromogen diaminobenzidine (DAB) staining (Cell Signaling Technology) and hematoxylin counter staining and mounted with xylene-based medium. We quantitatively scored the tissue slides under a microscope according to the percentage of positive cells and staining intensity. We assigned the following proportion scores: 0, 0% of cells being positive; 1, 0% to 1%; 2, 2% to 10%; 3, 11% to 30%; 4, 31% to 70%; and 5, 71% to 100%. We also rated the staining intensity on a scale of 0 to 3: 0, negative; 1, weak; 2, moderate; and 3, strong. The proportion and intensity scores were then combined by addition to obtain a total score (range 0–8), as described previously [18]. Two pathologists (X.F. and S.S.), who were blinded to the clinical information, independently validated the reproducibility of the scoring system.

### Statistical analysis

SPSS version 20.0 software (SPSS Inc., Chicago, IL, USA) was used for data analysis. *PGK1* and *PGK2* mRNA levels in tumor and normal tissues were compared using the independent variable *t* test. The associations between *PGK1* mRNA levels, PGK1 pS203 and PDHK1 pT338 levels and clinicopathologic characteristics of patients were analyzed using one-way analysis of variance (ANOVA) with the post hoc Bonferroni test for multiple comparisons and least significant difference test. The methylation value (M value) method is regarded as more statistically valid than the beta value method [19]. The correlation between the M values of the 11 probes covering the *PGK1* gene and *PGK1* mRNA levels was analyzed using Spearman’s correlation coefficient. The correlation between PGK1 pS203 and PDHK1 pT338 levels was analyzed using the Pearson correlation coefficient. Overall survival (OS) was defined as the duration from the date of diagnosis to death or the last known date of follow-up. The survival analyses were performed using the K-means cluster analysis to stratify the expression levels of related markers, the Kaplan–Meier method to plot survival curves, the log-rank test to compare survival rate, and a Cox regression model with two-sided Wald tests to calculate hazard ratios (HR) and 95% confidence intervals (CIs). Censored data were used for patients who were alive at last follow-up or lost to follow-up. Variables in univariate analysis with *P* values less than 0.05 were included in multivariate analysis. *P* < 0.05 was considered statistically significant. All statistical tests were two-sided.

## Results

### *PGK1* and *PGK2* expression in human cancers

**Fig. 1:**
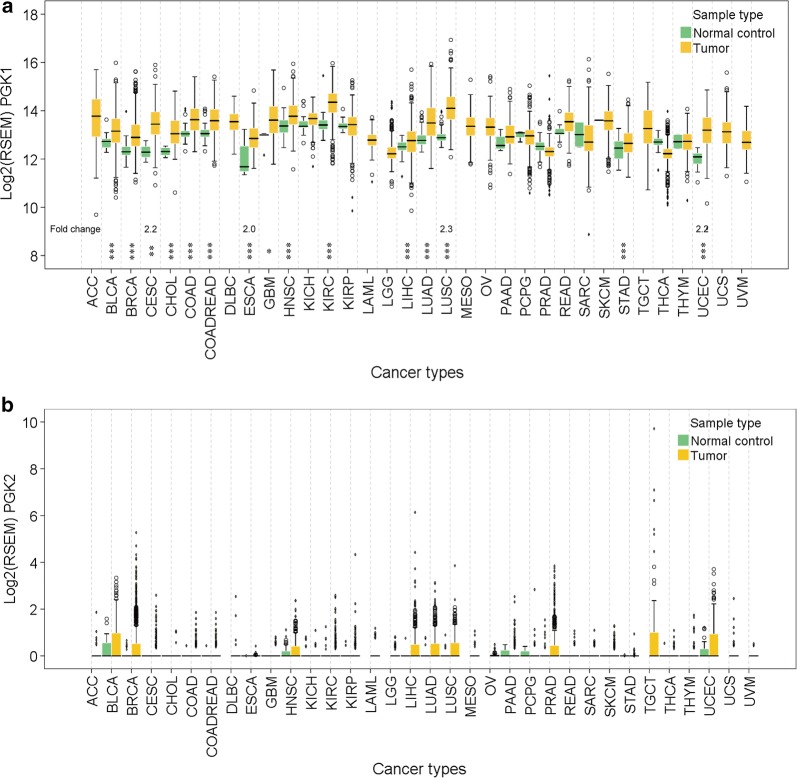
*PGK1* and *PGK2* mRNA levels in human cancer tissues and matched normal tissues. The data of *PGK1* (**a**) and *PGK2* (**b**) mRNA levels in human tumors and paired normal tissues were obtained from TCGA RNA-Seq datasets as log RSEM values (see “Materials and methods” section) and plotted. The fold changes of median *PGK1* mRNA level in tumors compared to that in normal controls in four cancer types are provided. Significant changes in median *PGK1* mRNA level between tumors and normal controls are marked with asterisks (*P < 0.05, **P < 0.01, ***P < 0.001, independent variable *t* test). *PGK1/2* phosphoglycerate kinase 1/2, *TCGA* The Cancer Genome Atlas, *ACC* adrenocortical cancer, *BLCA* bladder urothelial carcinoma, *BRCA* breast carcinoma, *CESC* cervical and endocervical cancer, *CHOL* cholangiocarcinoma, *COAD* colon adenocarcinoma, *COADREAD* colon and rectum adenocarcinoma, *DLBC* diffuse large B-cell lymphoma, *ESCA* esophageal carcinoma, *GBM* glioblastoma multiforme, *HNSC* head and neck squamous cell carcinoma, *KICH* kidney chromophobe, *KIRC* kidney clear cell carcinoma, *KIRP* kidney papillary cell carcinoma, *LAML* lymphoblastic acute myeloid leukaemia, *LGG* brain lower grade glioma, *LIHC* liver hepatocellular carcinoma, *LUAD* lung adenocarcinoma, *LUSC* lung squamous cell carcinoma, *MESO* mesothelioma, *OV* ovarian serous cystadenocarcinoma, *PAAD* pancreatic adenocarcinoma, *PCPG* pheochromocytoma and paraganglioma, *PRAD* prostate adenocarcinoma, *READ* rectum adenocarcinoma, *SARC* sarcoma, *SKCM* skin cutaneous melanoma, *STAD* stomach adenocarcinoma, *TGCT* testicular germ cell tumor, *THCA* thyroid carcinoma, *THYM* thymoma, *UCEC* uterine corpus endometrioid carcinoma, *UCS* uterine carcinosarcoma, *UVM* uveal melanoma

To compare *PGK1* and *PGK2* mRNA levels between tumor and normal tissues, we analyzed the RNA-Seq data of 11,908 tumor cases with 1582 paired normal tissues across 34 cancer types from TCGA datasets. In all types of cancer, the *PGK1* mRNA levels in tumor and matched normal tissues were approximately 2^12^ to 2^14^ times higher than those of *PGK2*, which could hardly be detected (Fig. 1). In addition, the data from the Illumina Body Map Project further confirmed that *PGK1*, which had the highest mRNA level in leukocytes, was the major isozyme in all tissues except in the testes (Additional file 1: Fig. S1).

Since nine cancer types did not have mRNA level data available for matched normal tissues, we analyzed 25 cancer types and found that 15 had significantly higher *PGK1* mRNA level in tumor tissues than in normal tissues and that *PGK1* mRNA levels were increased by more than twofolds in 4 of the 15 cancer types, namely, esophageal carcinoma (ESCA), uterine corpus endometrioid carcinoma (UCEC), cervical and endocervical cancer (CESC), and lung squamous cell carcinoma (LUSC) (Fig. 1a). In addition, the 15 cancer types account for 55.0% of cancer incidence and 63.1% of cancer mortality each year worldwide (data from GLOBOCAN 2018) [20], indicating that *PGK1* overexpression is prevalent among the most deadly human cancers.

### Association between *PGK1* mRNA levels and human cancer progression

**Fig. 2:**
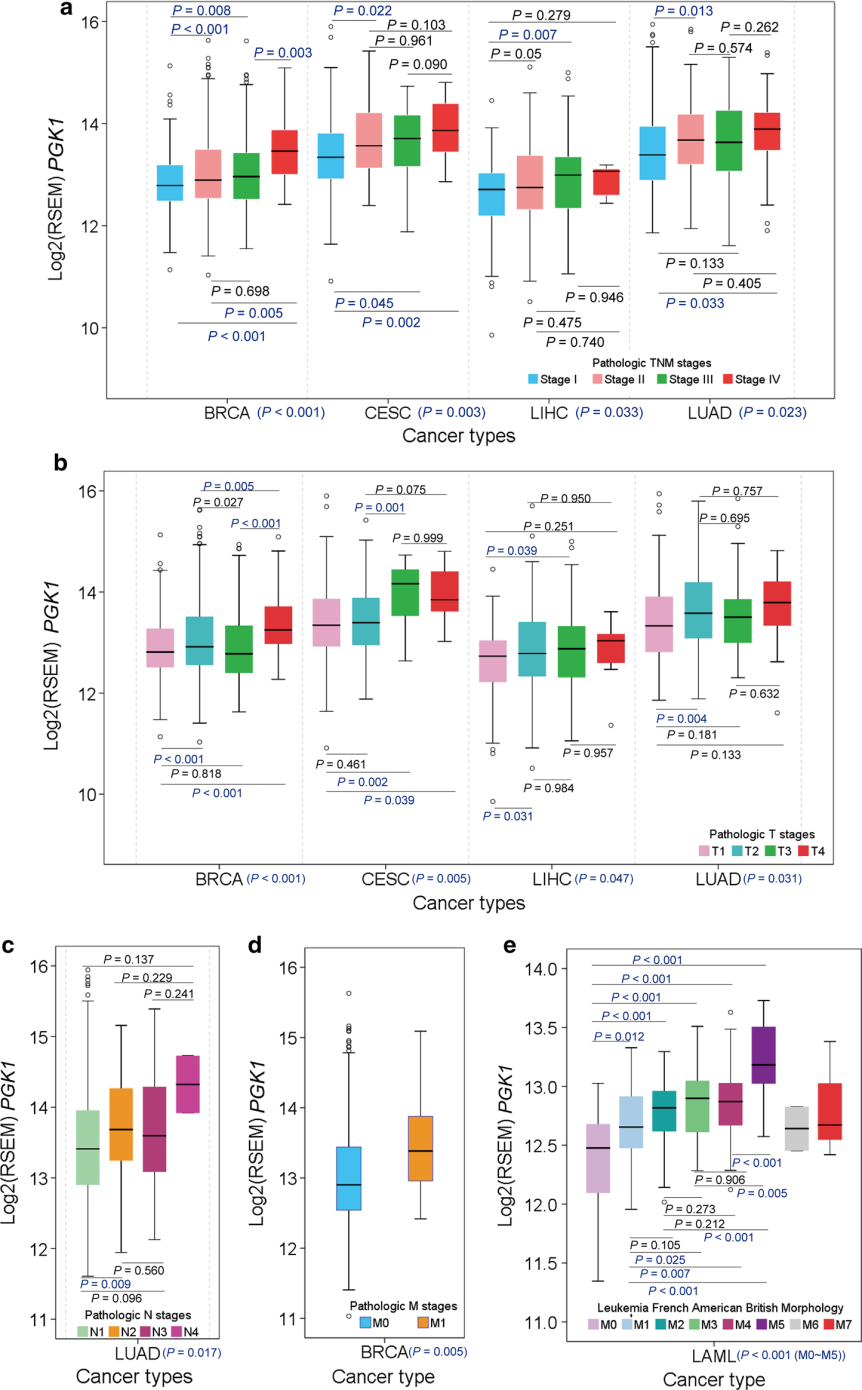
Association between *PGK1* mRNA levels and human cancer progression. **a** *PGK1* mRNA levels are associated with pathologic TNM stage of BRCA, CESC, LIHC, LUAD, and TGCT. **b**–**d** *PGK1* mRNA levels are associated were with T stage of BRCA, CESC, LIHC, and LUAD (**b**); N stage of LUAD and TGCT (**c**); and M stage of BRCA (**d**). **e** *PGK1* mRNA levels are associated with LAML M0 through M5. *BRCA* breast carcinoma, *CESC* cervical and endocervical cancer, *LIHC* liver hepatocellular carcinoma, *LUAD* lung adenocarcinoma, *TGCT* testicular germ cell tumor, *LAML* lymphoblastic acute myeloid leukaemia. The *P* values of the overall comparison between groups are presented on horizontal axes

To access whether the *PGK1* overexpression in tumor tissues was related to cancer progression, we analyzed the TNM staging data of the 15 cancer types with significantly increased *PGK1* mRNA levels. We found that *PGK1* mRNA level was significantly associated with progressive pathologic TNM stage in breast carcinoma (BRCA), CESC, liver hepatocellular carcinoma (LIHC), and lung adenocarcinoma (LUAD) (Fig. 2a). Further analyses showed that *PGK1* mRNA level was significantly associated with T stage in BRCA, CESC, LIHC, and LUAD (Fig. 2b); N stage in LUAD (Fig. 2c); and M stage in BRCA (Fig. 2d).

Lymphoblastic acute myeloid leukaemia (LAML) has eight subtypes, M0 through M7. Subtypes M0 through M5 have a high percentage of immature myeloblasts, with M0 myeloblasts appearing the least mature and M5 myeloblasts appearing the most mature histologically, whereas subtypes M6 and M7 have a high percentage of immature erythrocytes and megakaryocytes, respectively [21–25]. Among the 16 tissue types, leucocytes had the highest *PGK1* mRNA level (Additional file 1: Fig. S1). Notably, *PGK1* mRNA levels were associated with LAML M0 through M5 (Fig. 2e), suggesting the association between *PGK1* mRNA level and myeloblast maturity.

### Association between *PGK1* promoter hypomethylation and *PGK1* mRNA level elevation

DNA methylation regulates gene expression and is implicated in tumor progression and therapeutic response [26, 27]. We next determined the methylation status of the *PGK1* gene in 14 cancer types with significantly elevated *PGK1* mRNA levels in the TCGA data. GBM was not included in further comparison and association analysis because there were only two matched normal tissues with DNA methylation data available.

**Fig. 3:**
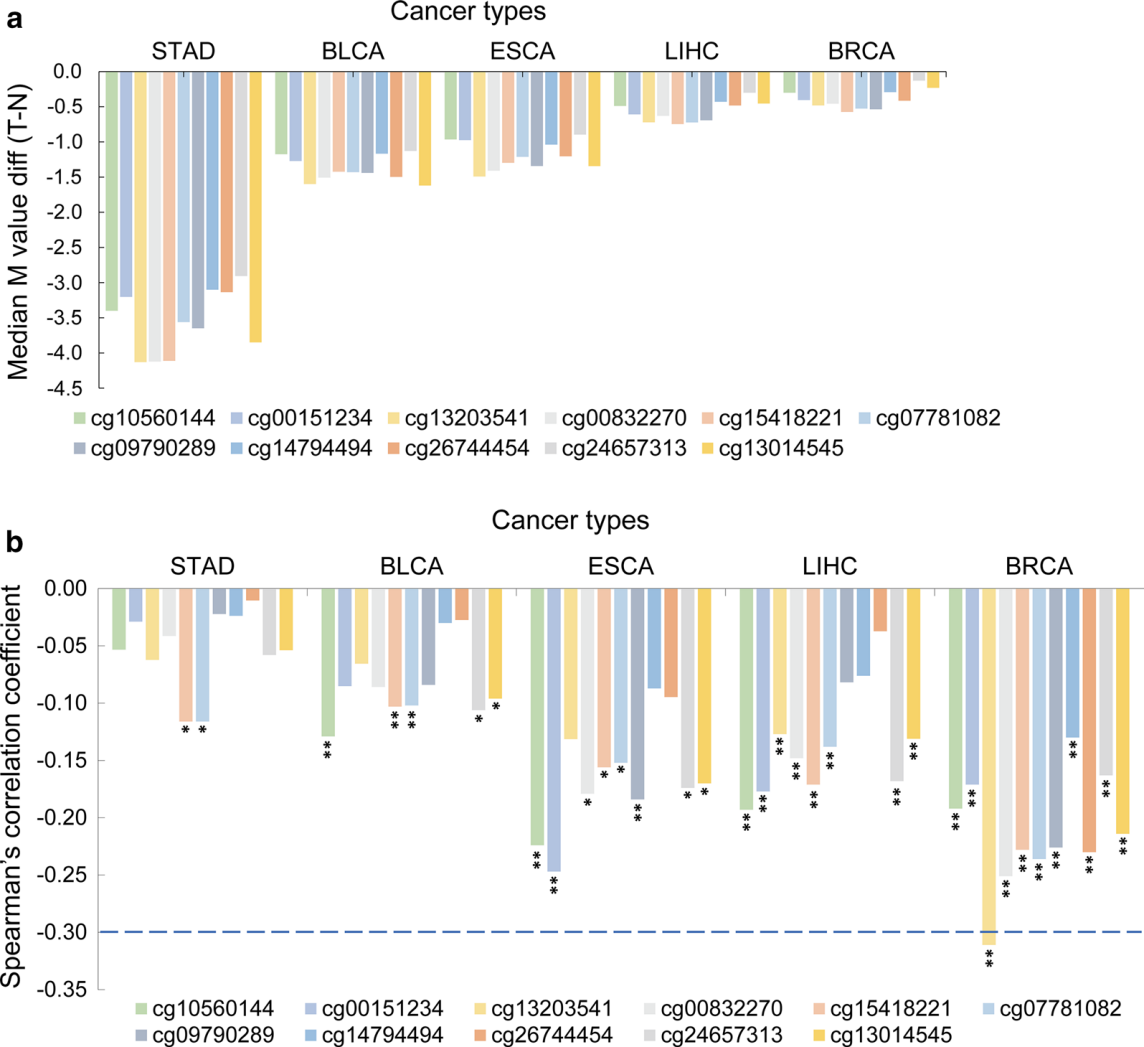
Association between *PGK1* promoter hypomethylation and *PGK1* mRNA level elevation in multiple cancer types. **a** All 11 methylation probes have unanimous values in five types of cancer, and these values are higher in normal tissues than in tumor tissues. *M value* the methylation level, *diff* difference, *T* − *N*, the methylation levels of probes in tumor tissues minus that in normal tissues. **b** *PGK1* promoter methylation is significantly associated with *PGK1* mRNA levels in STAD, BLCA, ESCA, LIHC, and BRCA (*P < 0.05, **P < 0.01, independent variable *t* test). All statistical tests were two-sided. *BRCA* breast carcinoma, *LIHC* liver hepatocellular carcinoma, *ESCA* esophageal carcinoma, *BLCA* bladder urothelial carcinoma, *STAD* stomach adenocarcinoma

Median differential M values between tumor and normal tissues were plotted in the 14 cancer types (Additional file 1: Fig. S2). We found that 11 methylation probes had unanimous values in five types of cancer [stomach adenocarcinoma (STAD), bladder urothelial carcinoma (BLCA), ESCA, LIHC, and BRCA], and these values were lower in tumor tissues than in normal tissues (Fig. 3a, Additional file 1: Fig. S2). Intriguingly, these 11 probes were all located in the *PGK1* promoter regions, ranging from 500 nt upstream of the transcription start site (TSS500) to the 5′-untranslated region (5′-UTR) (Additional file 1: Table S2; Additional file 1: Fig. S3), indicating that the *PGK1* promoter regions were hypomethylated in these five types of cancer.

The methylation data for these probes did not follow a normal distribution (1-sample Kolmogorov–Smirnov test, asymptotic *P* < 0.001, two-tailed; Additional file 1: Table S3). We identified a significant inverse correlation between the methylation levels and mRNA levels of *PGK1* in STAD, BLCA, ESCA, LIHC, and BRCA (Fig. 3b; Additional file 1: Table S4), suggesting promoter hypomethylation as a mechanism promoting *PGK1* expression. Among the five cancer types, BRCA showed the strongest correlation (Fig. 3b).

### Associations between *PGK1* promoter hypomethylation and *PGK1* mRNA level elevation and poor prognosis in BRCA patients

**Fig. 4:**
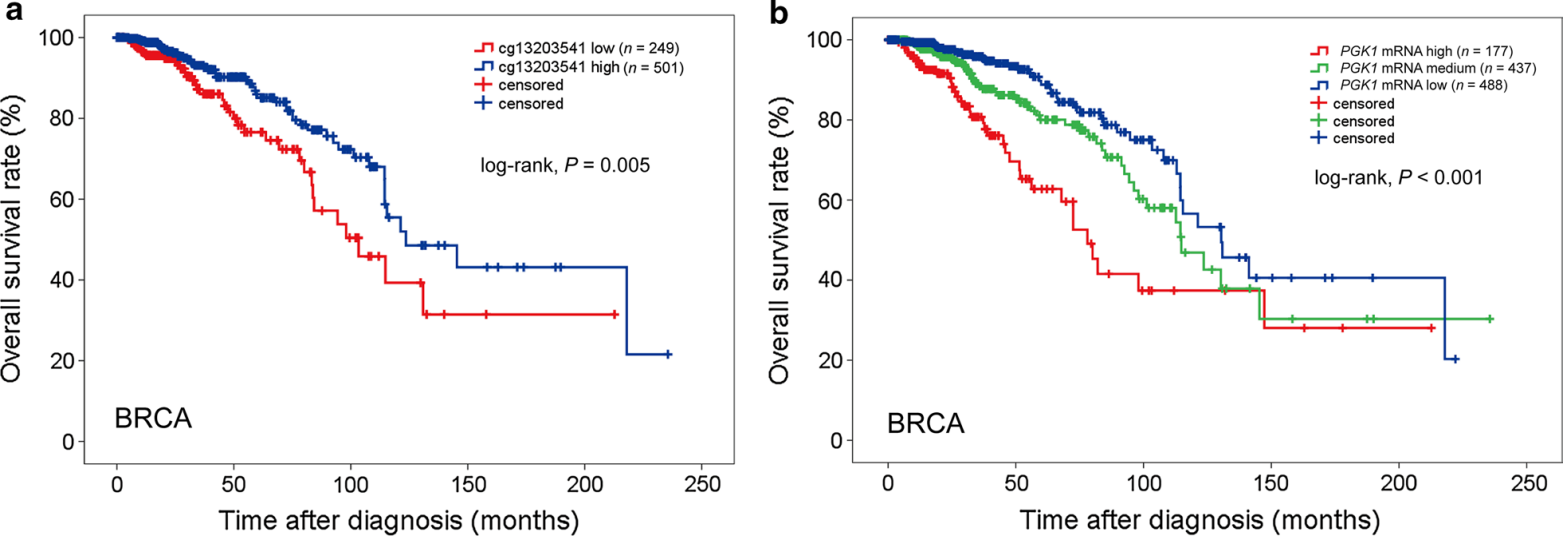
Associations of *PGK1* promoter hypomethylation and *PGK1* mRNA level with OS of BRCA patients. **a** High cg13203541 methylation levels are associated with prolonged OS of BRCA patients. **b** High *PGK1* mRNA levels are associated with short OS of BRCA patients. All statistical tests were two-sided. *OS* overall survival, *BRCA* breast carcinoma

We next analyzed the association between *PGK1* promoter hypomethylation and the survival of patients with STAD, BLCA, ESCA, LIHC, and BRCA, and found that only in BRCA, hypomethylation of cg13203541 was associated with short OS (HR = 0.551, 95% CI 0.361–0.841, *P* = 0.005; Additional file 1: Table S5; Fig. 4a). A multivariate Cox regression model showed that cg13203541 methylation was an independent predictor of prolonged OS in BRCA (HR = 0.599, 95% CI 0.382–0.939, *P* = 0.026; Additional file 1: Table S5). In line with these results, an inverse correlation between *PGK1* mRNA level and the OS of BRCA patients was also identified (HR = 1.966, 95% CI 1.535–2.519, *P* < 0.001; Fig. 4b). These results suggest that *PGK1* promoter methylation and mRNA level may be prognostic markers for BRCA patients.

### Correlation between elevated PGK1 pS203 and PDHK1 pT338 levels and their associations with cancer prognosis

**Fig. 5:**
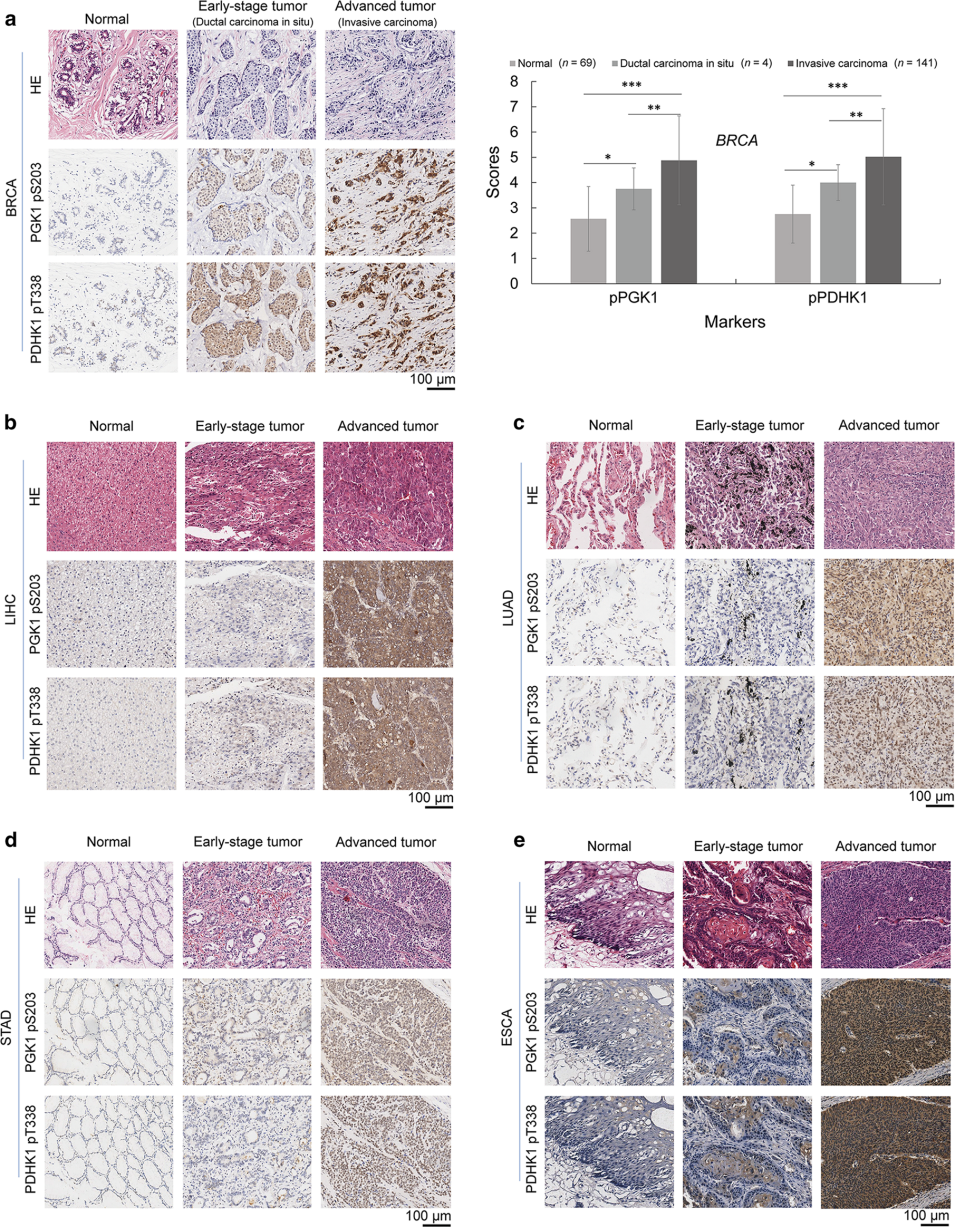
PGK1 pS203 and PDHK1 pT338 levels increased from normal to tumor tissues in human cancers. PGK1 pS203 and PDHK1 pT338 levels in normal tissues, early-stage tumor tissues, and advanced tumor tissues were compared using immunohistochemical staining. Representative images are shown. **a** Breast carcinoma (BRCA). **b** Liver hepatocellular carcinoma (LIHC). **c** Lung adenocarcinoma (LUAD). **d** Stomach adenocarcinoma (STAD). **e** Esophageal carcinoma (ESCA). *P < 0.05, **P < 0.01, ***P < 0.001 (2-tailed). *PGK1 pS203* phosphorylated phosphoglycerate kinase 1 (PGK1) S203, *PDHK1 pT338* phosphorylated phosphorylate pyruvate dehydrogenase kinase 1 (PDHK1) T338, *HE* hematoxylin–eosin staining

**Fig. 6:**
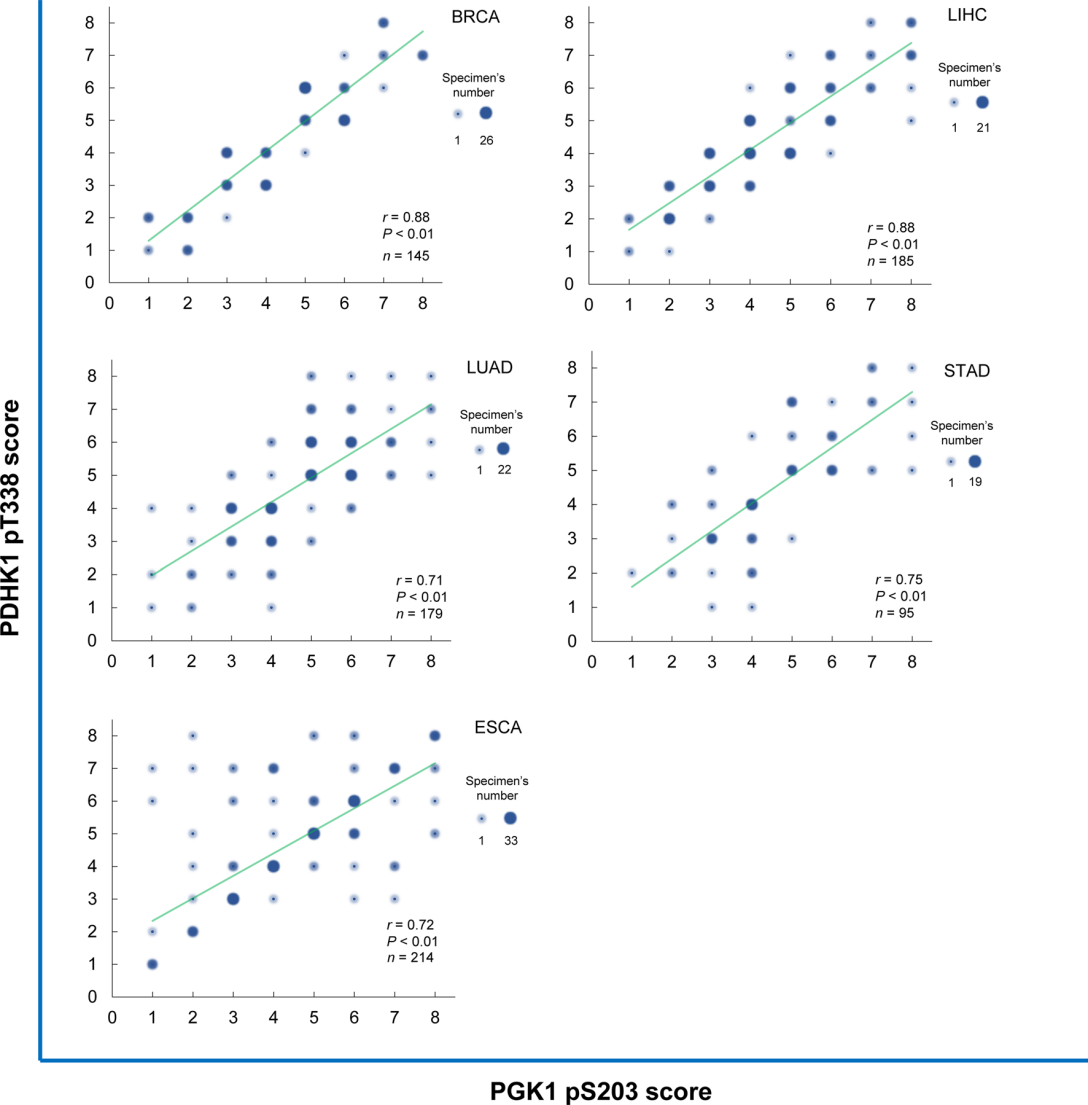
PGK1 pS203 and PDHK1 pT338 levels are positively correlated with each other in human cancers. Scatter diagrams show the statistical results of the correlation between PGK1 pS203 and PDHK1 pT338 levels (analyzed using the Pearson correlation coefficient). The size of each dot reflects the number of specimens. *PGK1 pS203* phosphorylated phosphoglycerate kinase 1 (PGK1) S203, *PDHK1 pT338* phosphorylated phosphorylate pyruvate dehydrogenase kinase 1 (PDHK1) T338; *BRCA* breast carcinoma, *LIHC* liver hepatocellular carcinoma, *LUAD* lung adenocarcinoma, *STAD* stomach adenocarcinoma, *ESCA* esophageal carcinoma

**Fig. 7:**
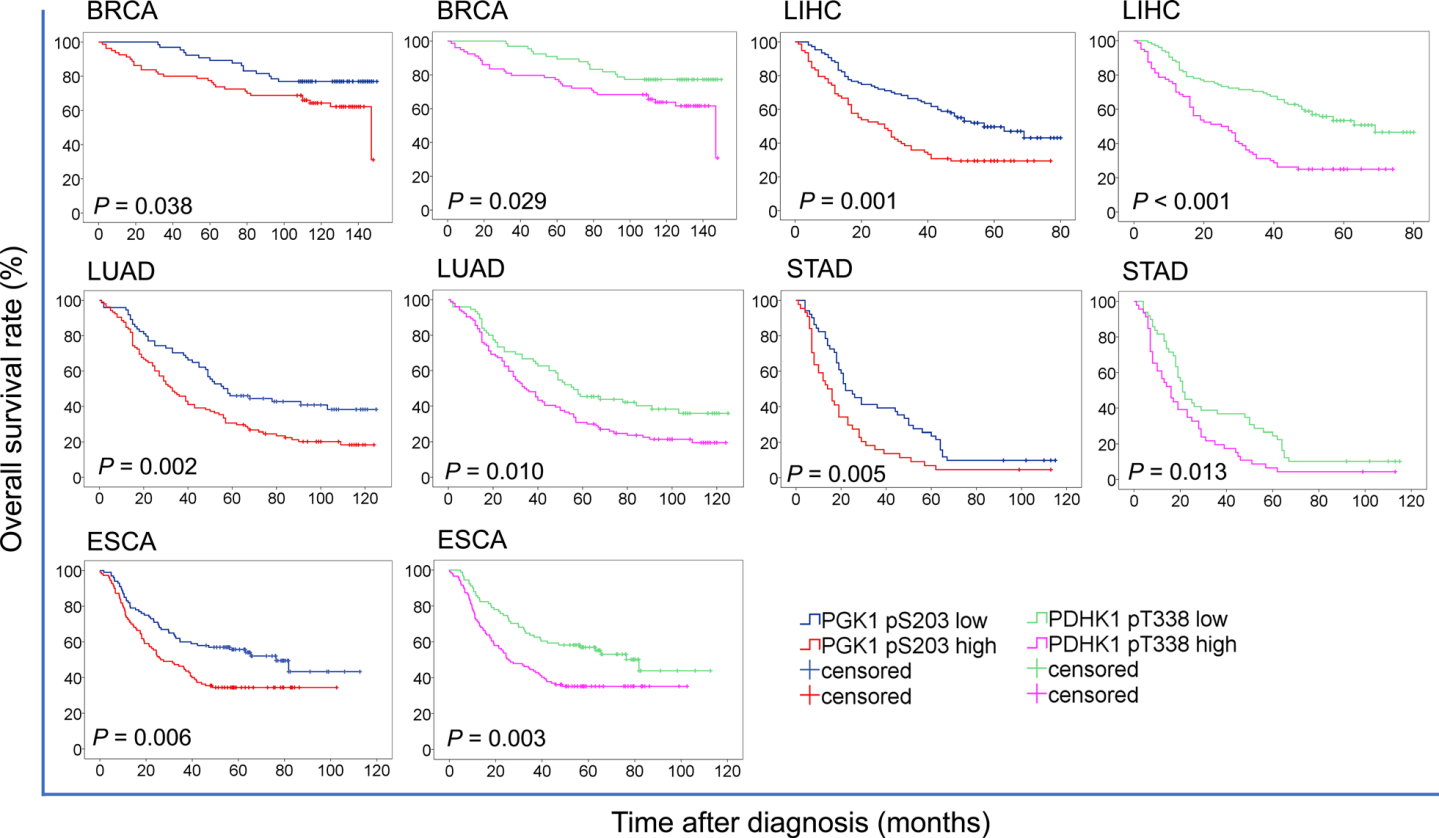
PGK1 pS203 and PDHK1 pT338 levels are associated with poor prognosis in cancer patients. K-Means cluster analysis was used to divide the indicated cancer patients into two groups with high and low levels of PGK1 pS203 and PDHK1 pT338. Kaplan–Meier survival curves were compared using the log-rank test. All statistical tests were two-sided. *PGK1 pS203* phosphorylated phosphoglycerate kinase 1 (PGK1) S203, *PDHK1 pT338* phosphorylated phosphorylate pyruvate dehydrogenase kinase 1 (PDHK1) T338, *BRCA* breast carcinoma, *LIHC* liver hepatocellular carcinoma, *LUAD* lung adenocarcinoma, *STAD* stomach adenocarcinoma, *ESCA* esophageal carcinoma

ERK-phosphorylated PGK1 S203 (PGK1 pS203) and PGK1-phosphorylated PDHK1 T338 (PDHK1 pT338) levels were found to be strongly correlated with each other and were both associated with GBM prognosis [14]. Here, we examined the phosphorylation levels of these two proteins in additional 818 independent cancer cases. We found that in all five cancer types, PGK1 pS203 and PDHK1 pT338 levels were higher in most tumor tissues than in their matched normal tissues (Additional file 1: Fig. S4), increasing from normal tissues to early-stage cancer tissues and to advanced carcinoma tissues (Fig. 5a–e), and positively correlating with each other in tumor tissues (Fig. 6). Kaplan–Meier analysis showed that higher levels of both PGK1 pS203 and PDHK1 pT338 were associated with shorter OS in patients with these five cancer types (all *P* < 0.05) (Fig. 7).

### Prognostic values of PGK1 pS203 and PDHK1 pT338 in cancer

An independent variable *t* test showed that both PGK1 pS203 and PDHK1 pT338 were associated with advanced TNM stage in patients with BRCA and ESCA (all *P* < 0.05) (Table 1). Univariate and multivariate Cox regression analyses showed that PGK1 pS203 was an independent predictor of short OS for LIHC (HR = 1.574, 95% CI 1.064–2.327, *P* = 0.023), LUAD (HR = 1.800, 95% CI 1.238–2.617, *P* = 0.002), and STAD (HR = 2.603, 95% CI 1.630–4.155, *P* < 0.001); PDHK1 pT338 was also an independent predictor of short OS for LIHC (HR = 2.060, 95% CI 1.390–3.052, *P* < 0.001), LUAD (HR = 1.634, 95% CI 1.129–2.364, *P* = 0.009), and STAD (HR = 2.397, 95% CI 1.501–3.829, *P* < 0.001) (Tables 2 and 3), suggesting that PGK1 phosphorylation and PGK1 protein kinase activity-mediated phosphorylation and activation of PDHK1 were instrumental for tumor progression and OS in multiple cancer types.

**Table 1:** Associations of PGK1 S203 and PDHK1 T338 phosphorylation levels with clinicopathologic characteristics in patients with BRCA, LIHC, LUAD, STAD and ESCA

Cancer type	Characteristic	Total (cases)	PGK1 pS203 (IHC staining score)			PDHK1 pT338 (IHC staining score)		
			Mean	95% CI	*P* value	Mean	95% CI	*P* value
BRCA	Age (years)				0.478			0.975
	≤ 60	96	4.70	4.34–5.06		4.99	4.60–5.38	
	> 60	49	4.92	4.41–5.42		5.00	4.47–5.53	
	TNM stage^a^				0.027			0.018
	I/II	95	4.54	4.18–4.90		4.73	4.34–5.11	
	III/IV	48	5.23	4.73–5.73		5.52	4.99–6.06	
LIHC	Age (years)							
	≤ 60	75	4.28	3.91–4.65	0.615	4.31	3.96–4.66	0.406
	> 60	110	4.42	4.06–4.78		4.52	4.19–4.85	
	Gender				0.138			0.365
	Male	159	4.44	4.17–4.71		4.47	4.22–4.72	
	Female	26	3.88	3.10–4.67		4.15	3.39–4.92	
	TNM stage^b^				0.643			0.226
	I/II	73	4.18	3.81–4.55		4.16	3.83–4.50	
	III/IV	104	4.30	3.95–4.64		4.46	4.13–4.79	
LUAD	Age (years)				0.763			0.385
	≤ 60	84	4.71	4.44–4.99		4.85	4.54–5.15	
	> 60	95	4.78	4.46–5.10		4.65	4.34–4.97	
	Gender				0.722			0.700
	Male	104	4.72	4.45–4.99		4.71	4.42–5.00	
	Female	75	4.79	4.45–5.14		4.79	4.45–5.14	
	TNM stage				0.339			0.236
	I/II	105	4.65	4.36–4.94		4.62	4.33–4.91	
	III/IV	74	4.86	4.54–5.18		4.89	4.56–5.22	
STAD	Age (years)				0.665			0.638
	≤ 60	56	4.55	4.18–4.93		4.61	4.21–5.01	
	> 60	39	4.69	4.14–5.24		4.44	3.82–5.05	
	Gender				0.149			0.593
	Male	60	4.78	4.39–5.18		4.47	4.01–4.92	
	Female	35	4.31	3.81–4.82		4.66	4.14–5.18	
	TNM stage^c^				0.356			0.195
	I/II	24	4.88	4.19–5.56		4.96	4.26–5.66	
	III/IV	69	4.54	4.17–4.90		4.45	4.06–4.84	
ESCA	Age (years)				0.781			0.089
	≤ 60	112	4.58	4.23–4.93		4.63	4.30–4.96	
	> 60	102	4.65	4.33–4.97		5.03	4.71–5.34	
	Gender				0.559			0.216
	Male	176	4.58	4.31–4.84		4.76	4.50–5.01	
	Female	38	4.76	4.23–5.29		5.13	4.56–5.70	
	TNM stage				0.006			0.008
	I/II	98	4.26	3.89–4.62		4.49	4.14–4.84	
	III/IV	116	4.91	4.61–5.22		5.1	4.81–5.40	

*BRCA* breast carcinoma, *LIHC* liver hepatocellular carcinoma, *LUAD* lung adenocarcinoma, *STAD* stomach adenocarcinoma, *ESCA* esophageal carcinoma, *95% CI* 95% confidence interval, *PGK1 pS203* PGK1 S203 phosphorylation level, *PDHK1 pT338* PDHK1 T338 phosphorylation level

^a^The data of TNM stage in BRCA were available in 143 patients

^b^The data of TNM stage in LIHC were available in 177 patients

^c^The data of TNM stage in STAD were available in 93 patients

**Table 2:** Univariate analyses of overall survival in additional independent cases of BRCA, LIHC, LUAD, STAD, and ESCA

Cancer type	Characteristic	Total (cases)	HR (95% CI)	*P*
BRCA	Age (years)			
	≤ 60	96	1.000	
	> 60	49	1.467 (0.864–2.752)	0.323
	TNBC^a^			
	No	109	1.000	
	Yes	24	2.296 (1.169–4.508)	0.016
	TNM stage^b^			
	I/II	95	1.000	
	III/IV	48	2.296 (1.279–4.120)	0.005
	PGK1 pS203			
	Low	65	1.000	
	High	80	1.905 (1.024–3.546)	0.042
	PDHK1 pT338			
	Low	66	1.000	
	High	79	1.969 (1.058–3.665)	0.032
LIHC	Age (years)			
	≤ 60	75	1.000	
	> 60	110	1.351 (0.863–1.962)	0.527
	Gender			
	Male	159	1.000	
	Female	26	0.597 (0.327–1.087)	0.092
	TNM stage^c^			
	I/II	73	1.000	
	III/IV	104	2.796 (1.848–4.229)	< 0.001
	PGK1 pS203			
	Low	107	1.000	
	High	78	1.909 (1.311–2.781)	0.001
	PDHK1 pT338			
	Low	105	1.000	
	High	80	2.354 (1.610–3.441)	< 0.001
LUAD	Age (years)			
	≤ 60	84	1.000	
	> 60	95	1.339 (0.944–1.900)	0.102
	Gender			
	Male	104	1.000	
	Female	75	0.775 (0.542–1.107)	0.162
	TNM stage			
	I/II	105	1.000	
	III/IV	74	1.861 (1.309–2.647)	0.001
	PGK1 pS203			
	Low	74	1.000	
	High	105	1.760 (1.221–2.537)	0.002
	PDHK1 pT338			
	Low	75	1.000	
	High	104	1.595 (1.111–2.291)	0.011
STAD	Age (years)			
	≤ 60	56	1.000	
	> 60	39	1.754 (1.144–2.689)	0.010
	Gender			
	Male	60	1.000	
	Female	35	1.017 (0.660–1.567)	0.938
	TNM stage^d^			
	I/II	24	1.000	
	III/IV	69	2.590 (1.536–4.365)	< 0.001
	PGK1 pS203			
	Low	51	1.000	
	High	44	1.797 (1.173–2.752)	0.007
	PDHK1 pT338			
	Low	49	1.000	
	High	46	1.694 (1.105–2.596)	0.016
ESCA	Age (years)			
	≤ 60	112	1.000	
	> 60	102	1.869 (1.300–2.686)	0.001
	Gender			
	Male	176	1.000	
	Female	38	1.127 (0.709–1.789)	0.614
	TNM stage			
	I/II	98	1.000	
	III/IV	116	6.447 (4.139–10.043)	< 0.001
	PGK1 pS203			
	Low	103	1.000	
	High	111	1.669 (1.157–2.406)	0.006
	PDHK1 pT338			
	Low	94	1.000	
	High	120	1.763 (1.213–2.563)	0.003

*BRCA* breast carcinoma, *LIHC* liver hepatocellular carcinoma, *LUAD* lung adenocarcinoma, *STAD* stomach adenocarcinoma, *ESCA* esophageal carcinoma, *HR* hazard ratio, *95% CI* 95% confidence interval, *TNBC* triple-negative breast cancer, *PGK1 pS203* phosphorylated phosphoglycerate kinase 1 (PGK1) S203, *PDHK1 pT338* phosphorylated phosphorylate pyruvate dehydrogenase kinase 1 (PDHK1) T338

^a^The data of TNBC in BRCA were available in 133 patients

^b^The data of TNM stage in BRCA were available in 143 patients

^c^The data of TNM stage in LIHC were available in 177 patients

^d^The data of TNM stage in STAD were available in 93 patients

**Table 3:** Multivariate analyses of overall survival in additional independent cases of BRCA, LIHC, LUAD, STAD, and ESCA

Cancer type	Characteristic	Total (cases)	PGK1 pS203		PDHK1 pT338	
			HR (95% CI)	*P*	HR (95% CI)	*P*
BRCA	TNBC^a^					
	No	109	1.000		1.000	
	Yes	24	2.561 (1.205–5.444)	0.014	2.532 (1.191 to 5.383)	0.016
	TNM stage^b^					
	I/II	95	1.000		1.000	
	III/IV	48	2.241 (1.172–4.286)	0.015	2.238 (1.174 to 4.265)	0.014
	PGK1 pS203					
	Low	65	1.000			
	High	80	1.596 (0.805–3.168)	0.181		
	PDHK1 pT338					
	Low	66			1.000	
	High	79			1.661 (0.837 to 3.298)	0.147
LIHC	TNM stage^c^					
	I/II	73	1.000		1.000	
	III/IV	104	2.853 (1.878 to 4.335)	0.000	2.905 (1.910 to 4.419)	0.000
	PGK1 pS203					
	Low	107	1.000			
	High	78	1.574 (1.064 to 2.327)	0.023		
	PDHK1 pT338					
	Low	105			1.000	
	High	80			2.060 (1.390 to 3.052)	0.000
LUAD	TNM stage					
	I/II	105	1.000		1.000	
	III/IV	74	1.805 (1.267 to 2.570)	0.001	1.828 (1.284 to 2.602)	0.001
	PGK1 pS203					
	Low	74	1.000			
	High	105	1.800 (1.238 to 2.617)	0.002		
	PDHK1 pT338					
	Low	75			1.000	
	High	104			1.634 (1.129 to 2.364)	0.009
STAD	Age (years)					
	≤ 60	56	1.000		1.000	
	> 60	39	2.258 (1.441 to 3.538)	0.000	2.255 (1.436 to 3.540)	0.000
	TNM stage^d^					
	I/II	24	1.000		1.000	
	III/IV	69	3.382 (1.957 to 5.843)	0.000	3.314 (1.920 to 5.720)	0.000
	PGK1 pS203					
	Low	51	1.000			
	High	44	2.603 (1.630 to 4.155)	0.000		
	PDHK1 pT338					
	Low	49			1.000	
	High	46			2.397 (1.501 to 3.829)	0.000
ESCA	Age (years)					
	≤ 60	112	1.000		1.000	
	> 60	102	1.657 (1.146 to 2.397)	0.007	1.614 (1.114 to 2.338)	0.011
	TNM stage					
	I/II	98	1.000		1.000	
	III/IV	116	6.041 (3.867 to 9.437)	0.000	6.022 (3.852 to 9.415)	0.000
	PGK1 pS203					
	Low	103	1.000			
	High	111	1.440 (0.997 to 2.079)	0.052		
	PDHK1 pT338					
	Low	94			1.000	
	High	120			1.453 (0.996 to 2.119)	0.053

*BRCA* breast carcinoma, *LIHC* liver hepatocellular carcinoma, *LUAD* lung adenocarcinoma, *STAD* stomach adenocarcinoma, *ESCA* esophageal carcinoma, *HR* hazard ratio, *95% CI* 95% confidence interval, *TNBC* triple-negative breast cancer, *HER2* human epidermal growth factor receptor 2, *PGK1 pS203* PGK1 S203 phosphorylation level, *PDHK1 pT338* PDHK1 T338 phosphorylation level

^a^The data of TNBC in BRCA were available in 133 patients

^b^The data of TNM stage in BRCA were available in 143 patients

^c^The data of TNM stage in LIHC were available in 177 patients

^d^The data of TNM stage in STAD were available in 93 patients

## Discussion

In the present study, we identified relationships of high *PGK1* mRNA level and promoter hypomethylation with advanced TNM stage and short OS in multiple cancer types in a pan-cancer analysis of TCGA data involving 11,908 cases covering 34 cancer types. Additional analyses of a cohort of 818 cases revealed that the phosphorylation levels of PGK1 S203 and PDHK1 T338 were independent prognostic biomarkers for LIHC, LUAD, and STAD. All these findings suggest that *PGK1* gene modification and PGK1-mitochondrial function were significantly associated with clinical behaviors of cancer patients.

Metabolic reprogramming plays an important role in tumorigenesis [4, 28–32]. It is emerging that the nonmetabolic functions of metabolic enzymes are fundamental to tumorigenesis [33]. We reported that the protein kinase activity of metabolic enzymes, such as PGK1 [14, 15, 34], pyruvate kinase M2 (PKM2) [35, 36], and ketohexokinase isoform A (KHK-A) [37], regulates the Warburg effect, gene expression, cell proliferation, and autophagy [28, 29].We previously found that mitochondrial PGK1 functions as a protein kinase to promote tumor cell proliferation and brain tumorigenesis [14]. In the present study, we investigated the clinical relevance of PGK1 in human cancers and found that elevated *PGK1* mRNA level and PGK1 protein kinase activity were associated with advanced TNM stages and poor prognosis in multiple human cancers. Whether other metabolic enzymes with protein kinase activity can act as potential biomarkers for the prediction of progression and prognosis of human cancers should be further analyzed.

The up-regulation of PGK1 involved in the Warburg effect has been detected in several types of human cancer [9–13]. Several other studies also reported the relationship between PGK1 acetylation and its innate enzymatic activity [38, 39]. However, all these studies focused on the metabolic function of PGK1 without elucidating the relationship between cancer progression and gene modification and the mitochondrial function of PGK1. Importantly, recent studies showed that metabolic changes in cancer alter the epigenetic landscape, especially DNA modifications, leading to malignant transformation, adaptation to inadequate nutrition, and tumor development [40]. Therefore, in the present study, we analyzed the DNA methylation data for 14 cancer types from TCGA datasets and identified hypomethylation of the *PGK1* promoter (cg13203541) as an independent prognostic biomarker in BRCA patients (Additional file 1: Table S5). We also detected mitochondrial PGK1-dependent PDHK1 T338 phosphorylation in additional cases of five cancer types and demonstrated that mitochondrial function of PGK1 significantly affected the clinical behaviors of patients with these cancers.

Reprogrammed energy metabolism is an emerging hallmark of cancer biology and is an important way to treat cancer [4, 29–31, 41]. One important example is isocitrate dehydrogenase 1 (IDH1) mutation, which has important clinical significance and was found in GBM [42] and myeloid malignancies, such as acute myelocytic leukaemia (AML) [43] and myelodysplastic syndromes (MDS) [44]. A clinical study suggested that IDH1 mutation was an independent, favorable prognostic marker in grade 2–4 glioma [45]. Related clinical trials are ongoing in AML [43, 44]. In the present study, we found another metabolism reprogramming mediated by PGK1 protein kinase activity-dependent phosphorylation, which was associated with clinical behaviors of cancer patients and was an independent prognostic biomarker in multiple types of cancer. In addition, we revealed a high percentage of patients exhibiting elevated protein kinase activity of PGK1 compared to a relatively low IDH1 mutations in cancer patients [42, 43, 45]. Thus, we underscore that PGK1 protein kinase activity is a potential target for cancer treatment.

Our research has several limitations. First, the number of cases of some cancer types were rather limited in TCGA datasets. For example, only 36 tumor samples of cholangiocarcinoma and 66 tumor samples of kidney chromophobe were identified. Second, data of normal samples were not available in several cancer types, therefore, some analyses could not be performed for these cancer types. Third, validation of the association of PGK1 phosphorylation with clinicopathological characteristics could only be made in independent Chinese cohorts covering five cancer types as there were few publicly available datasets regarding the phosphorylation levels of proteins, and we could not validate those associations in Caucasians tumor samples.

## Conclusions

We demonstrated a relationship between *PGK1* promoter methylation and *PGK1* mRNA level and demonstrated the significance of *PGK1* mRNA level, *PGK1* promoter methylation, and PGK1 pS203 and PDHK1 pT338 levels in tumor progression and cancer patient survival. These findings highlight the potential use of *PGK1* mRNA level, *PGK1* promoter hypomethylation, and PGK1 pS203 and PDHK1 pT338 levels as biomarkers for cancer progression and prognosis, and the promising significance of PGK1 as a target in cancer treatment.

## Supplementary information

**Additional file 1.** Additional methods, Figures S1–S4 and Tables S1–S5.

## Data Availability

The data generated or analyzed during this study are included in this article or are available from the corresponding author upon reasonable request.

## Abbreviations

5′-UTR: 5′-untranslated region
95% CI: 95% confidence interval
ACC: adrenocortical cancer
ADP: adenosine diphosphate
ANOVA: analysis of variance
ATP: adenosine triphosphate
BLCA: bladder urothelial carcinoma
BRCA: breast carcinoma
CESC: cervical and endocervical cancer
CHOL: cholangiocarcinoma
COAD: colon adenocarcinoma
COADREAD: colorectal adenocarcinoma
DLBC: diffuse large B cell lymphoma
ESCA: esophageal carcinoma
GBM: glioblastoma multiforme
GDC: Genomic Data Commons
H&E: Mayer’s haematoxylin and eosin
HNSC: head and neck squamous cell carcinoma
HR: hazard ratio
KICH: kidney chromophobe
KIRC: kidney clear cell carcinoma
KIRP: kidney papillary cell carcinoma
LAML: lymphoblastic acute myeloid leukaemia
LGG: brain lower grade glioma
LIHC: liver hepatocellular carcinoma
LUAD: lung adenocarcinoma
LUSC: lung squamous cell carcinoma
MESO: mesothelioma
OV: ovarian serous cystadenocarcinoma
PAAD: pancreatic adenocarcinoma
PCPG: pheochromocytoma and paraganglioma
PDHK1: pyruvate dehydrogenase kinase 1
PGK1: phosphoglycerate kinase 1
PIN1: peptidyl-prolyl *cis*–*trans* isomerase NIMA-interacting 1
PRAD: prostate adenocarcinoma
READ: rectum adenocarcinoma
RSEM: RNA-Seq by Expectation Maximization
SARC: sarcoma
SKCM: skin cutaneous melanoma
STAD: stomach adenocarcinoma
TCGA: The Cancer Genome Atlas
TGCT: testicular germ cell tumor
THCA: thyroid carcinoma
THYM: thymoma
TOM: translocase of the outer membrane
TPM: transcripts per million
TSS500: 500 nt upstream of the transcription start site
UCEC: uterine corpus endometrioid carcinoma
UCS: uterine carcinosarcoma
UCSC: University of California, Santa Cruz
UVM: uveal melanoma
