# Supplementary material for: Associations of PGK1 promoter hypomethylation and PGK1-mediated PDHK1 phosphorylation with cancer stage and prognosis: a TCGA pan-cancer analysis
Source: Cancer Commun (Lond). 2019 Oct 2;39:54. doi: 10.1186/s40880-019-0401-9 (PMC6775656; doi:10.1186/s40880-019-0401-9)
Supplement: Supplementary file 1 — Additional file 1. Additional methods, Figures S1–S4 and Tables S1–S5. [file 40880_2019_401_MOESM1_ESM.docx]

**Additional Methods**

**Detailed TCGA data**

The gene level profile was measured experimentally using the Illumina HiSeq 2000 RNA Sequencing platform produced by The Cancer Genome Atlas (TCGA) genome characterization center (data producer) in the University of North Carolina. Level 3 data was downloaded from TCGA data coordination center (https://nitaac.nih.gov/resources/testimonials-and-whitepapers/case-studies/cancer-genome-atlas-data-coordinating-center-and). This dataset shows the gene-level transcription estimates, as in log2(x+1) transformed RNA-Seq by Expectation Maximization (RSEM) normalized count. Genes are mapped onto the human genome coordinates using the University of California, Santa Cruz (UCSC) Xena Human Genome Organisation (HUGO) probeMap. The detailed methods can be referred to method description from University of North Carolina TCGA genome characterization center: Data Coordinating Center (DCC) description (https://xenabrowser.net/datapages/).

DNA methylation profile was measured using the Illumina Infinium HumanMethylation450 platform. Beta values were derived at the Johns Hopkins University and University of South California TCGA genome characterization center. DNA methylation values, described as beta values, are recorded for each array probe in each sample via BeadStudio software (Illumina, Inc.; San Diego, CA, USA). DNA methylation beta values are continuous variables between 0.0 and 1.0, representing the ratio of the intensity of the methylated bead type to the combined locus intensity. Thus, higher beta values represent higher level of DNA methylation, i.e. hypermethylation, and lower beta values represent lower level of DNA methylation, i.e. hypomethylation. We observed a bimodal distribution of beta values from both methylation27 and methylation450 platforms, with two peaks around 0.1 and 0.9 and a relatively flat valley around 0.2-0.8. The bimodal distribution is far more pronounced and balanced in the methylation450 platform than in the methylation27 platform. In the methylation27 platform, the lower beta peak is much stronger than the higher beta peak, while the two peaks are of similar height in the methylation450 platform. Microarray probes are mapped onto the human genome coordinates using xena probeMap derived from GEO GPL13534 record. Here is a reference to Illumina Infinium BeadChip DNA methylation platform beta value (https://xenabrowser.net/datapages/).

**Additional Tables**

**Table S1. Summary of the sample sizes for *PGK1* RNA-Seq and DNA methylation data across different cancer types from TCGA datasets.**

| **Cancer type** | **RNA sequencing (cases)** | | **DNA methylation (cases)** | |
| --- | --- | --- | --- | --- |
|  | **Normal control** | **Tumor** | **Normal control** | **Tumor** |
| Adrenocortical cancer (ACC) | 0 | 79 | 0 | 0 |
| Bladder urothelial carcinoma (BLCA) | 19 | 407 | 21 | 413 |
| Breast carcinoma (BRCA) | 113 | 1102 | 98 | 774 |
| Cervical & endocervical cancer (CESC) | 3 | 305 | 3 | 309 |
| Cholangiocarcinoma (CHOL) | 9 | 36 | 9 | 36 |
| Colon adenocarcinoma (COAD) | 41 | 288 | 38 | 299 |
| Colorectal adenocarcinoma (COADREAD) | 50 | 383 | 45 | 388 |
| Diffuse large B-cell lymphoma (DLBC) | 0 | 48 | 0 | 0 |
| Esophageal carcinoma (ESCA) | 11 | 185 | 16 | 186 |
| Glioblastoma multiforme (GBM) | 5 | 167 | 2 | 153 |
| Head & neck squamous cell carcinoma (HNSC) | 43 | 521 | 50 | 530 |
| Kidney chromophobe (KICH) | 25 | 66 | 0 | 0 |
| Kidney clear cell carcinoma (KIRC) | 72 | 534 | 160 | 320 |
| Kidney papillary cell carcinoma (KIRP) | 32 | 291 | 0 | 0 |
| Lymphoblastic acute myeloid leukemia (LAML) | 0 | 173 | 0 | 0 |
| Brain lower grade glioma (LGG) | 0 | 530 | 0 | 0 |
| Liver hepatocellular carcinoma (LIHC) | 50 | 373 | 50 | 379 |
| Lung adenocarcinoma (LUAD) | 58 | 513 | 32 | 460 |
| Lung squamous cell carcinoma (LUSC) | 51 | 502 | 43 | 372 |
| Mesothelioma (MESO) | 0 | 87 | 0 | 0 |
| Ovarian serous cystadenocarcinoma (OV) | 0 | 308 | 0 | 0 |
| Pancreatic adenocarcinoma (PAAD) | 4 | 179 | 0 | 0 |
| Pheochromocytoma & paraganglioma (PCPG) | 3 | 184 | 0 | 0 |
| Prostate adenocarcinoma (PRAD) | 52 | 498 | 0 | 0 |
| Rectum adenocarcinoma (READ) | 9 | 95 | 0 | 0 |
| Sarcoma (SARC) | 2 | 262 | 0 | 0 |
| Skin cutaneous melanoma (SKCM) | 1 | 472 | 0 | 0 |
| Stomach adenocarcinoma (STAD) | 35 | 415 | 2 | 396 |
| Testicular germ cell tumor (TGCT) | 0 | 156 | 0 | 0 |
| Thyroid carcinoma (THCA) | 59 | 513 | 0 | 0 |
| Thymoma (THYM) | 2 | 119 | 0 | 0 |
| Uterine corpus endometrioid carcinoma (UCEC) | 24 | 175 | 46 | 432 |
| Uterine carcinosarcoma (UCS) | 0 | 57 | 0 | 0 |
| Uveal melanoma (UVM) | 0 | 80 | 0 | 0 |

Abbreviations: TCGA, The Cancer Genome Atlas; PGK1, phosphoglycerate kinase 1.

All data are presented as the number of specimens in related datasets.

**Table S2. Comprehensive information on all 16 probes of the Illumina Infinium Human Methylation 450 BeadChip array covering the *PGK1* gene.**

| Illumina probe ID | Genome build | MI | Source sequence | Strand | UCSC gene group |
| --- | --- | --- | --- | --- | --- |
| cg11887015 | 37 | 77359083 | GCAATGTAATTTTTCTGGTTATGGCATGATTGATGCTAATGGCAAACACG | F | TSS1500 |
| cg10560144 | 37 | 77359355 | ATTCCACGGGGTTGGGGTTGCGCCTTTTCCAAGGCAGCCCTGGGTTTGCG | R | TSS1500 |
| cg00151234 | 37 | 77359363 | GGGTTGGGGTTGCGCCTTTTCCAAGGCAGCCCTGGGTTTGCGCAGGGACG | R | TSS1500 |
| cg13203541 | 37 | 77359450 | GGAGCAGGAAGCGTCGCCGGGGGGCCCACAAGGGTAGCGGCGAAGATCCG | F | TSS1500 |
| cg00832270 | 37 | 77359535 | TGCGAGGGTACTAGTGAGACGTGCGGCTTCCGTTTGTCACGTCCGGCACG | F | TSS200 |
| cg15418221 | 37 | 77359544 | CTGTCCGTCTGCGAGGGTACTAGTGAGACGTGCGGCTTCCGTTTGTCACG | F | TSS200 |
| cg07781082 | 37 | 77359553 | CGGAAGCCGCACGTCTCACTAGTACCCTCGCAGACGGACAGCGCCAGGGA | F | TSS200 |
| cg09790289 | 37 | 77359560 | CGGCTTCCGTTTGTCACGTCCGGCACGCCGCGAACCGCAAGGAACCTTCC | R | TSS200 |
| cg14794494 | 37 | 77359564 | GCTGCCATTGCTCCCTGGCGCTGTCCGTCTGCGAGGGTACTAGTGAGACG | F | TSS200 |
| cg26744454 | 37 | 77359613 | GTCTCACTAGTACCCTCGCAGACGGACAGCGCCAGGGAGCAATGGCAGCG | R | TSS200 |
| cg24657313 | 37 | 77359739 | CGCGGGCAGGAACAGGGCCCACACTACCGCCCCACACCCCGCCTCCCGCA | R | 1stExon; 5'-UTR |
| cg13014545 | 37 | 77359749 | CGGAACACCGCGCGGGCAGGAACAGGGCCCACACTACCGCCCCACACCCC | R | 1stExon; 5'-UTR |
| cg16181613 | 37 | 77359860 | CCTCTCTCCCCAGCTGTATTTCCAAAATGTCGCTTTCTAACAAGCTGACG | R | 1stExon |
| cg20203466 | 37 | 77359924 | AACAAGTCGGCTCCGGCCAAAGAGGTTTGCGACAGAGCACAGAGAGCACG | F | 1stIntron; Body |
| cg08955859 | 37 | 77360510 | TATGTCACAATCCTGATGCAAATCCTGTGCCCAAGAGTCCAACGATACCG | F | Body |
| cg18989233 | 37 | 77381829 | CGGATCAGATGTCTATATTGCTGAATGCAAGAAGTGGGGCAGCAGCAGTG | F | 3'-UTR |

Abbreviations: PGK1, phosphoglycerate kinase 1; MI, map information; UCSC, University of California, Santa Cruz; TSS, transcription start site; UTR, untranslated region.

**Table S3. Distribution of methylation data for 11 probes covering the *PGK1* gene in STAD, BLCA, ESCA, LIHC, and BRCA.**

| **Cancer type** | **total (cases)** | **Statistic** | | **Methylation probe** | | | | | | | | | | |
| --- | --- | --- | --- | --- | --- | --- | --- | --- | --- | --- | --- | --- | --- | --- |
|  |  |  |  | cg10560144 | cg00151234 | cg13203541 | cg00832270 | cg15418221 | cg07781082 | cg09790289 | cg14794494 | cg26744454 | cg24657313 | cg13014545 |
| **STAD** | 398 | Normal parameter | Mean | -3.783 | -3.571 | -3.851 | -4.109 | -3.927 | -4.190 | -4.053 | -3.503 | -4.088 | -4.676 | -4.110 |
|  |  |  | SD | 2.185 | 2.218 | 2.833 | 2.988 | 2.803 | 2.649 | 2.684 | 2.297 | 2.437 | 2.202 | 2.877 |
|  |  | Most extreme difference | Absolute | 0.336 | 0.318 | 0.316 | 0.346 | 0.348 | 0.350 | 0.346 | 0.300 | 0.307 | 0.323 | 0.335 |
|  |  |  | Positive | 0.336 | 0.318 | 0.316 | 0.346 | 0.348 | 0.350 | 0.346 | 0.300 | 0.307 | 0.323 | 0.335 |
|  |  |  | Negative | -0.164 | -0.177 | -0.198 | -0.213 | -0.189 | -0.186 | -0.191 | -0.191 | -0.177 | -0.180 | -0.191 |
|  |  | Kolmogorov-Smirnov Z | | 6.698 | 6.344 | 6.310 | 6.895 | 6.944 | 6.978 | 6.899 | 5.967 | 6.129 | 6.441 | 6.679 |
|  |  | Asymptotic ***P*** (2-tailed) | | <0.001 | <0.001 | <0.001 | <0.001 | <0.001 | <0.001 | <0.001 | <0.001 | <0.001 | <0.001 | <0.001 |
| **BLCA** | 434 | Normal parameter | Mean | -4.085 | -3.922 | -4.287 | -4.600 | -4.431 | -4.659 | -4.495 | -3.909 | -4.427 | -5.126 | -4.604 |
|  |  |  | SD | 2.020 | 1.998 | 2.605 | 2.740 | 2.564 | 2.398 | 2.446 | 2.093 | 2.228 | 1.967 | 2.643 |
|  |  | Most extreme difference | Absolute | 0.329 | 0.330 | 0.329 | 0.367 | 0.364 | 0.353 | 0.372 | 0.308 | 0.331 | 0.338 | 0.338 |
|  |  |  | Positive | 0.329 | 0.330 | 0.329 | 0.367 | 0.364 | 0.353 | 0.372 | 0.308 | 0.331 | 0.338 | 0.338 |
|  |  |  | Negative | -0.180 | -0.180 | -0.180 | -0.210 | -0.205 | -0.200 | -0.209 | -0.160 | -0.191 | -0.197 | -0.197 |
|  |  | Kolmogorov-Smirnov Z | | 6.848 | 6.878 | 6.853 | 7.646 | 7.593 | 7.346 | 7.745 | 6.365 | 6.891 | 7.045 | 7.033 |
|  |  | Asymptotic ***P*** (2-tailed) | | <0.001 | <0.001 | <0.001 | <0.001 | <0.001 | <0.001 | <0.001 | <0.001 | <0.001 | <0.001 | <0.001 |
| **ESCA** | 202 | Normal parameter | Mean | -4.552 | -4.396 | -4.973 | -5.258 | -4.958 | -5.165 | -5.081 | -4.485 | -5.042 | -5.523 | -5.236 |
|  |  |  | SD | 1.729 | 1.763 | 2.243 | 2.356 | 2.190 | 2.051 | 2.115 | 1.829 | 1.948 | 1.698 | 2.269 |
|  |  | Most extreme difference | Absolute | 0.381 | 0.362 | 0.385 | 0.397 | 0.383 | 0.390 | 0.415 | 0.352 | 0.410 | 0.368 | 0.380 |
|  |  |  | Positive | 0.381 | 0.362 | 0.385 | 0.397 | 0.383 | 0.390 | 0.415 | 0.352 | 0.410 | 0.368 | 0.380 |
|  |  |  | Negative | -0.225 | -0.227 | -0.230 | -0.260 | -0.251 | -0.248 | -0.244 | -0.209 | -0.217 | -0.243 | -0.247 |
|  |  | Kolmogorov-Smirnov Z | | 5.411 | 5.138 | 5.474 | 5.649 | 5.439 | 5.540 | 5.895 | 4.976 | 5.830 | 5.234 | 5.394 |
|  |  | Asymptotic ***P*** (2-tailed) | | <0.001 | <0.001 | <0.001 | <0.001 | <0.001 | <0.001 | <0.001 | <0.001 | <0.001 | <0.001 | <0.001 |
| **LIHC** | 429 | Normal parameter | Mean | -3.853 | -3.773 | -3.902 | -4.322 | -4.168 | -4.418 | -4.283 | -3.746 | -4.154 | -4.968 | -4.267 |
|  |  |  | SD | 2.062 | 1.990 | 2.746 | 2.809 | 2.548 | 2.400 | 2.494 | 2.078 | 2.389 | 1.973 | 2.773 |
|  |  | Most extreme difference | Absolute | 0.328 | 0.324 | 0.326 | 0.346 | 0.334 | 0.340 | 0.349 | 0.303 | 0.331 | 0.296 | 0.304 |
|  |  |  | Positive | 0.328 | 0.324 | 0.326 | 0.346 | 0.334 | 0.340 | 0.349 | 0.303 | 0.331 | 0.296 | 0.304 |
|  |  |  | Negative | -0.176 | -0.172 | -0.179 | -0.193 | -0.179 | -0.181 | -0.187 | -0.159 | -0.177 | -0.180 | -0.182 |
|  |  | Kolmogorov-Smirnov Z | | 6.785 | 6.711 | 6.758 | 7.169 | 6.920 | 7.047 | 7.229 | 6.256 | 6.852 | 6.127 | 6.303 |
|  |  | Asymptotic ***P*** (2-tailed) | | <0.001 | <0.001 | <0.001 | <0.001 | <0.001 | <0.001 | <0.001 | <0.001 | <0.001 | <0.001 | <0.001 |
| **BRCA** | 872 | Normal parameter | Mean | -0.752 | -0.650 | -0.023 | -0.213 | -0.316 | -0.837 | -0.587 | -0.678 | -0.820 | -2.378 | -0.375 |
|  |  |  | SD | 0.845 | 0.840 | 0.844 | 0.768 | 0.877 | 0.914 | 0.810 | 0.776 | 0.756 | 1.130 | 0.824 |
|  |  | Most extreme difference | Absolute | 0.168 | 0.165 | 0.198 | 0.197 | 0.172 | 0.154 | 0.182 | 0.133 | 0.203 | 0.112 | 0.211 |
|  |  |  | Positive | 0.113 | 0.099 | 0.159 | 0.173 | 0.122 | 0.109 | 0.135 | 0.133 | 0.146 | 0.084 | 0.157 |
|  |  |  | Negative | -0.168 | -0.165 | -0.198 | -0.197 | -0.172 | -0.154 | -0.182 | -0.129 | -0.203 | -0.112 | -0.211 |
|  |  | Kolmogorov-Smirnov Z | | 4.968 | 4.875 | 5.861 | 5.807 | 5.066 | 4.542 | 5.385 | 3.918 | 5.980 | 3.302 | 6.231 |
|  |  | Asymptotic ***P*** (2-tailed) | | <0.001 | <0.001 | <0.001 | <0.001 | <0.001 | <0.001 | <0.001 | <0.001 | <0.001 | <0.001 | <0.001 |

Abbreviations: PGK1, phosphoglycerate kinase 1; STAD, stomach adenocarcinoma; BLCA, bladder urothelial carcinoma; ESCA, esophageal carcinoma; LIHC, liver hepatocellular carcinoma; BRCA, breast carcinoma; SD, standard deviation.

**Table S4. Correlations between the methylation levels of 11 probes and *PGK1* mRNA levels in STAD, BLCA, ESCA, LIHC, and BRCA.**

| ***PGK1* probe** | **Parameter** | **Cancer type** | | | | |
| --- | --- | --- | --- | --- | --- | --- |
|  |  | **STAD** | **BLCA** | **ESCA** | **LIHC** | **BRCA** |
| cg10560144 | *n* | 377 | 424 | 196 | 414 | 855 |
|  | Spearman’s *r* | -0.053 | -0.129** | -0.224** | -0.193** | -0.192** |
|  | ***P*** (2-tailed) | 0.302 | 0.008 | 0.002 | < 0.001 | < 0.001 |
|  |  |  |  |  |  |  |
| cg00151234 | *n* | 377 | 424 | 196 | 414 | 855 |
|  | Spearman’s *r* | -0.029 | -0.085 | -0.247** | -0.177** | -0.171** |
|  | ***P*** (2-tailed) | 0.577 | 0.080 | < 0.001 | < 0.001 | < 0.001 |
| cg13203541 | *n* | 377 | 424 | 196 | 414 | 855 |
|  | Spearman’s *r* | -0.062 | -0.066 | -0.131 | -0.127** | -0.311** |
|  | ***P*** (2-tailed) | 0.228 | 0.178 | 0.066 | 0.010 | < 0.001 |
|  |  |  |  |  |  |  |
| cg00832270 | *n* | 377 | 424 | 196 | 414 | 855 |
|  | Spearman’s *r* | -0.041 | -0.086 | -0.179* | -0.148** | -0.251** |
|  | ***P*** (2-tailed) | 0.423 | 0.077 | 0.012 | 0.003 | < 0.001 |
|  |  |  |  |  |  |  |
| cg15418221 | *n* | 377 | 424 | 196 | 414 | 855 |
|  | Spearman’s *r* | -0.116* | -0.103* | -0.156* | -0.171** | -0.228** |
|  | ***P*** (2-tailed) | 0.024 | 0.035 | 0.029 | < 0.001 | < 0.001 |
|  |  |  |  |  |  |  |
| cg07781082 | *n* | 377 | 424 | 196 | 414 | 855 |
|  | Spearman’s *r* | -0.116* | -0.102* | -0.152* | -0.138** | -0.236** |
|  | ***P*** (2-tailed) | 0.024 | 0.035 | 0.034 | 0.005 | < 0.001 |
|  |  |  |  |  |  |  |
| cg09790289 | *n* | 377 | 424 | 196 | 414 | 855 |
|  | Spearman’s *r* | -0.022 | -0.084 | -0.184** | -0.082 | -0.226** |
|  | ***P*** (2-tailed) | 0.667 | 0.084 | 0.010 | 0.097 | < 0.001 |
|  |  |  |  |  |  |  |
| cg14794494 | *n* | 374 | 418 | 194 | 411 | 854 |
|  | Spearman’s *r* | -0.024 | -0.030 | -0.087 | -0.076 | -0.130** |
|  | ***P*** (2-tailed) | 0.647 | 0.540 | 0.227 | 0.124 | < 0.001 |
|  |  |  |  |  |  |  |
| cg26744454 | *n* | 377 | 424 | 196 | 414 | 855 |
|  | Spearman’s *r* | -0.010 | -0.027 | -0.095 | -0.037 | -0.230** |
|  | ***P*** (2-tailed) | 0.840 | 0.573 | 0.187 | 0.450 | < 0.001 |
|  |  |  |  |  |  |  |
| cg24657313 | *n* | 377 | 424 | 196 | 414 | 855 |
|  | Spearman’s *r* | -0.058 | -0.106* | -0.174* | -0.168** | -0.163** |
|  | ***P*** (2-tailed) | 0.261 | 0.029 | 0.015 | < 0.001 | < 0.001 |
|  |  |  |  |  |  |  |
| cg13014545 | *n* | 377 | 424 | 196 | 414 | 855 |
|  | Spearman’s *r* | -0.054 | -0.096* | -0.170* | -0.131** | -0.214** |
|  | ***P*** (2-tailed) | 0.298 | 0.048 | 0.018 | 0.008 | < 0.001 |
|  |  |  |  |  |  |  |

Abbreviations: PGK1, phosphoglycerate kinase 1; BLCA, bladder urothelial carcinoma; BRCA, breast carcinoma; ESCA, esophageal carcinoma; LIHC, liver hepatocellular carcinoma; STAD, stomach adenocarcinoma.

**. Correlation is significant at the 0.01 level (2-tailed).

*. Correlation is significant at the 0.05 level (2-tailed).

**Table S5. Cox univariate and multivariable analyses of overall survival of BRCA patients.**

| **Characteristic**  **(BRCA)** | **Total (cases)** | **Univariate analysis** | |  | **Multivariable analysis** | |
| --- | --- | --- | --- | --- | --- | --- |
|  |  | **HR (95% CI)** | ***P*** |  | **HR (95% CI)** | ***P*** |
| *PGK1* mRNA |  |  |  |  |  |  |
| Low | 463 | 1.000 |  |  | 1.000 |  |
| High | 268 | 2.364 (1.544-3.618) | < 0.001 |  | 2.453 (1.560-3.855) | < 0.001 |
| TNM stage |  |  |  |  |  |  |
| I and II | 535 | 1.000 |  |  | 1.000 |  |
| III and IV | 196 | 1.758 (1.129-2.737) | 0.013 |  | 1.819 (1.165-2.839) | 0.009 |
| cg13203541 methylation |  |  |  |  |  |  |
| Low | 233 | 1.000 |  |  | 1.000 |  |
| High | 498 | 0.551 (0.360-0.843) | 0.006 |  | 0.599 (0.382-0.939) | 0.026 |

Abbreviations: PGK1, phosphoglycerate kinase 1; BRCA, breast carcinoma; HR, hazard ratio; 95% CI, 95% confidence interval. .

**Additional Figures**

**
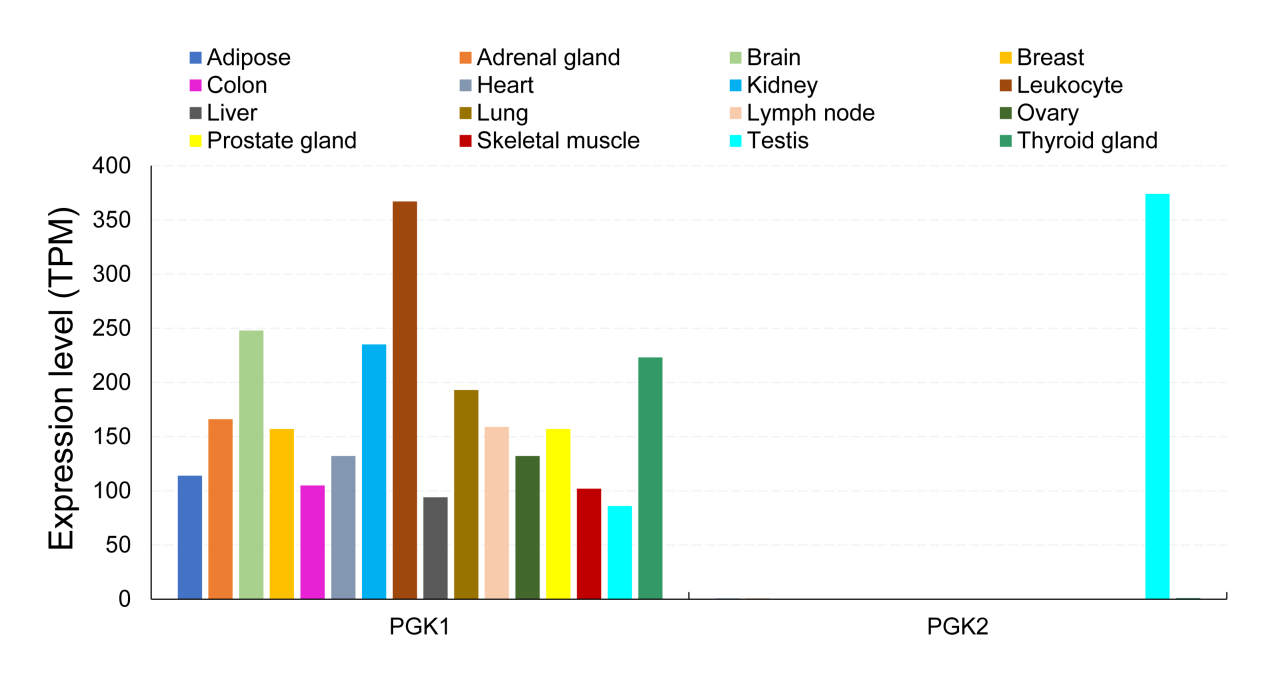
**

**Fig. S1. *PGK1* and *PGK2* mRNA** levels **in 16 types of normal tissues.** Data were obtained from the Illumina Body Map Project (https://www.ebi.ac.uk/gxa/home). TPM, transcripts per million; PGK1/2, phosphoglycerate kinase 1/2.


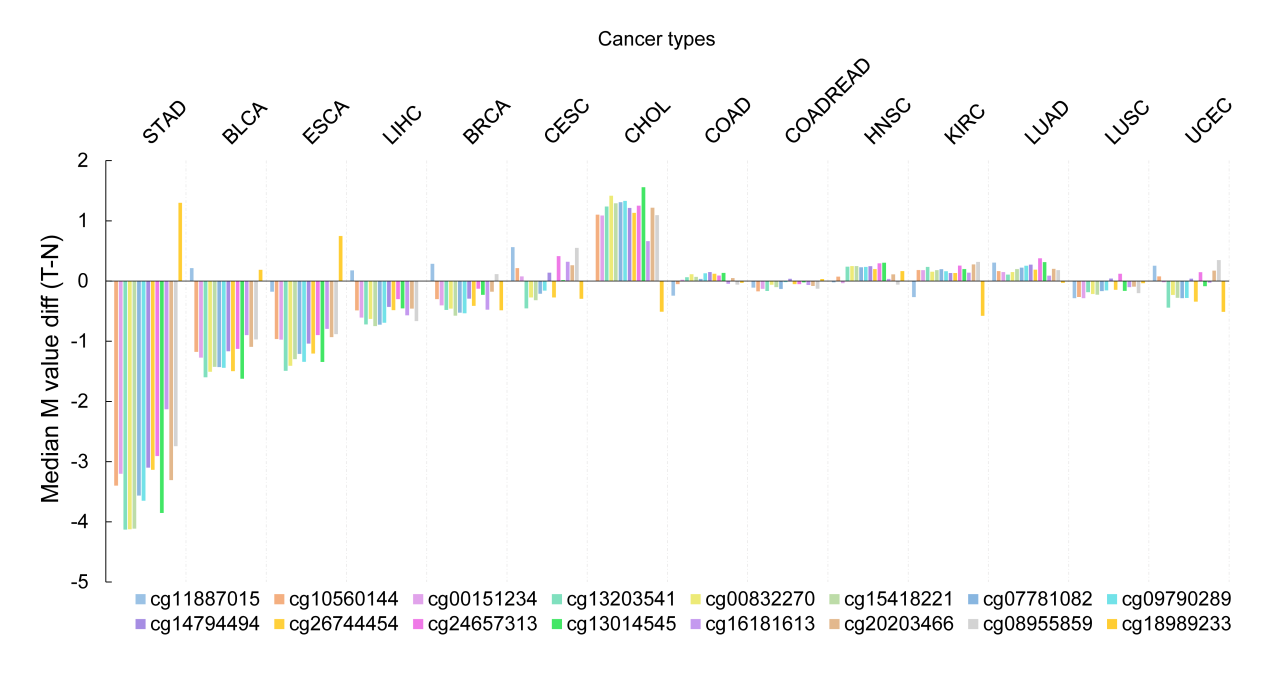


**Fig. S2. Median differential M values of 16 probes covering the *PGK1* gene between tumor and normal control tissues in 14 cancer types.** All these cancer types have significantly increased PGK1 mRNA levels in tumor tissues compared with those in normal tissues. M value, the methylation level; diff, difference; T-N, the methylation levels of probes in tumor tissues minus that in normal tissues.


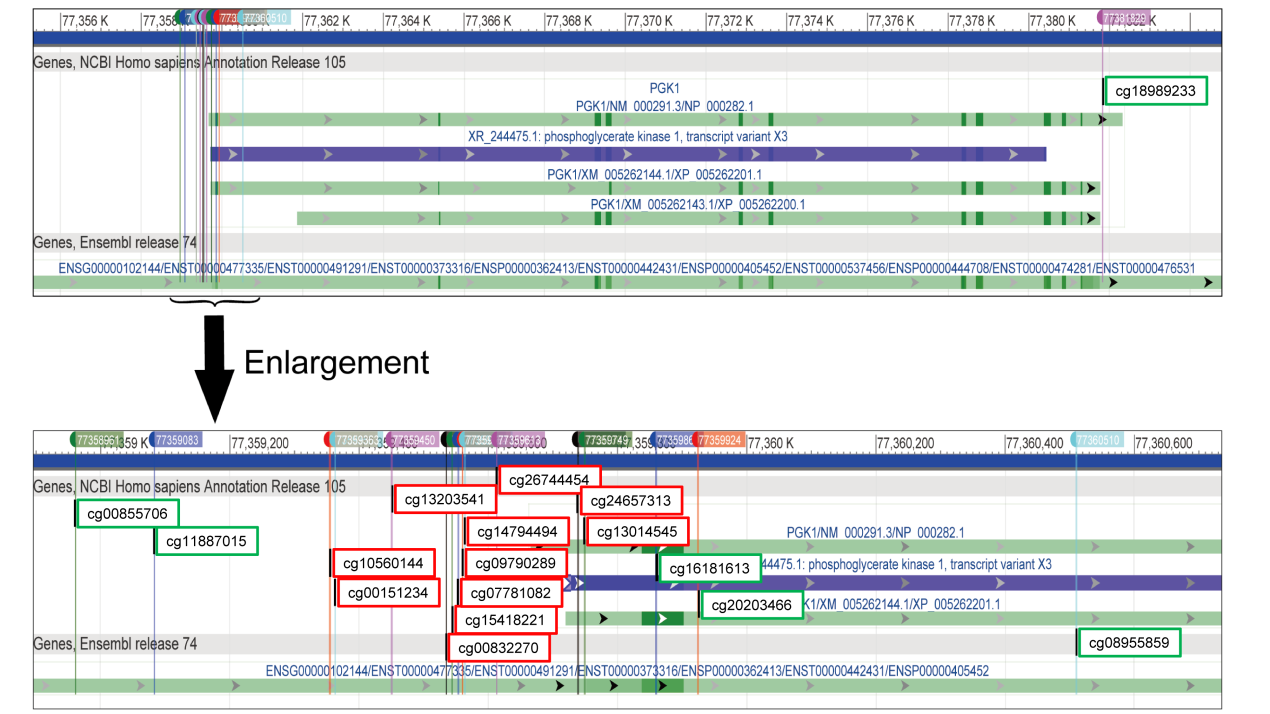


**Fig. S3. *PGK1* gene structure and the positions of probes in the Illumina Infinium Human Methylation 450 BeadChip array covering the *PGK1* gene.** Eleven probes (orange box) are located in the *PGK1* promoter region.


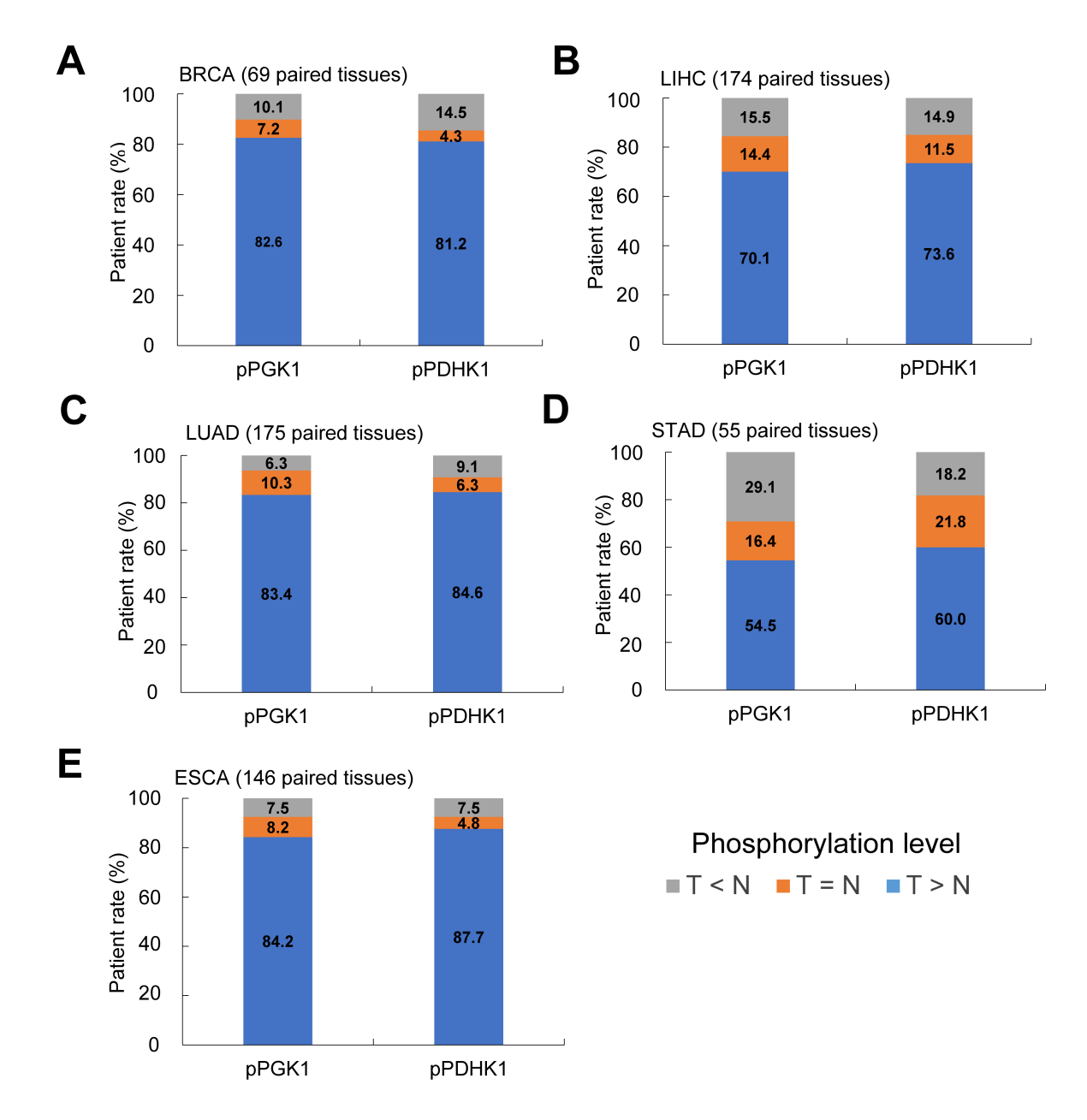


**Fig. S4. PGK1 pS203 and PDHK1 pT338 levels in tumor and matched normal tissues using immunohistochemical staining in BRCA, LIHC, LUAD, STAD, and ESCA.** **A,** Breast carcinoma (BRCA). **B,** Liver hepatocellular carcinoma (LIHC). **C,** Lung adenocarcinoma (LUAD). **D,** Stomach adenocarcinoma (STAD). **E,** Esophageal carcinoma (ESCA).

Abbreviations: T, tumor tissue; N, normal tissue; pPGK1, phosphorylated phosphoglycerate kinase 1 (PGK1) S203; pPDHK1, phosphorylated phosphorylate pyruvate dehydrogenase kinase 1 (PDHK1) T338.
